# Regulation of biofilm formation in *Zymomonas mobilis* to enhance stress tolerance by heterologous expression of *pfs* and *luxS*

**DOI:** 10.3389/fbioe.2023.1130405

**Published:** 2023-02-08

**Authors:** Lian-Ying Cao, Chen-Guang Liu, Shi-Hui Yang, Feng-Wu Bai

**Affiliations:** ^1^ State Key Laboratory of Microbial Metabolism, Joint International Research Laboratory of Metabolic & Developmental Science, School of Life Sciences and Biotechnology, Shanghai Jiao Tong University, Shanghai, China; ^2^ State Key Laboratory of Biocatalysis and Enzyme Engineering, School of Life Sciences, Hubei University, Wuhan, China

**Keywords:** *Zymomonas mobilis*, quorum sensing, *pfs*, *luxS*, biofilm, stress tolerance

## Abstract

*Zymomonas mobilis* is a potential alternative of *Saccharomyces cerevisiae* to produce cellulosic ethanol with strengths in cofactor balance, but its lower tolerance to inhibitors in the lignocellulosic hydrolysate restricts the application. Although biofilm can improve bacteria stress tolerance, regulating biofilm formation in *Z. mobilis* is still a challenge. In this work, we constructed a pathway by heterologous expressing *pfs* and *luxS* from *Escherichia coli* in *Z. mobili*s to produce AI-2 (autoinducer 2), a universal quorum-sensing signal molecule, to control cell morphology for enhancing stress tolerance. Unexpectedly, the results suggested that neither endogenous AI-2 nor exogenous AI-2 promoted biofilm formation, while heterologous expression of *pfs* can significantly raise biofilm. Therefore, we proposed that the main factor in assisting biofilm formation was the product accumulated due to heterologous expression of *pfs*, like methylated DNA. Consequently, ZM4::pfs produced more biofilm, which presented an enhanced tolerance to acetic acid. All these findings provide a novel strategy to improve the stress tolerance of *Z. mobilis* by enhancing biofilm formation for efficient production of lignocellulosic ethanol and other value-added chemical products.

## 1 Introduction

*Zymomonas mobilis* is a promising strain in lignocellulosic ethanol production because of its rapid usage of glucose through the Entner–Doudoroff pathway and its superior ability to efficiently utilize pentose by expressing four genes encoding xylose assimilation and pentose phosphate pathway enzymes from *Escherichia coli* (Zhang et al., 1995; Ming et al., 2014). The toxic byproducts from lignocellulosic hydrolysate significantly impair the growth and viability of *Z. mobilis* (Franden et al., 2013), thus limits its application in industrial fermentation. Several traits, such as adaptive laboratory evolution, error-prone PCR-based whole genome shuffling, transposon-based random mutagenesis, omics mining, and molecular manipulation, have been successfully exploited to strengthen the tolerance of *Z. mobilis* to specific inhibitors (Yang et al., 2010a; Yang et al., 2010b; Jia et al., 2013; Shui et al., 2015; Wang et al., 2016; Huang et al., 2018). However, the requirement for tolerance to multiple inhibitors in lignocellulosic hydrolysate is still a challenge.

Biofilm is an ordered structure composed of microorganisms, extracellular polysaccharide substance (EPS), protein, and extracellular DNA, which could immobilize microorganisms within the bioreactor to improve fermentation efficiency (Todhanakasem et al., 2019; Xia et al., 2019). Studies have shown that biofilm formation of *Z. mobilis* can significantly enhance the tolerance to various inhibitors compared with planktonic cells (Todhanakasem, 2016; Todhanakasem et al., 2019). Biotic or abiotic carriers such as corn silk, DEAE-cellulose, polystyrene, and polyvinyl chloride have been employed as platforms for *Z. mobilis* to generate biofilm with improved fermentation efficiency (Todhanakasem, 2016; Todhanakasem et al., 2019).

Quorum sensing (QS) is an intercellular communication triggered by a high dosage of specific organic molecules, usually causes acute morphological or physiological changes of bacteria such as bioluminescence, virulence factor secretion, biofilm formation, and biofilm dispersion (Whiteley et al., 2017). For decades, several sorts of QS signal molecules have been identified, including AI-2 (autoinducer-2), AHLs (acyl-homoserine lactone), DSFs (diffusible signal molecules), and AIP (autoinducing peptide) (Papenfort and Bassler, 2016). Among them, AI-2 is the most widespread QS signal molecule that exists in both Gram-positive and Gram-negative bacteria, mediates inter-species and intra-species communication (Pereira et al., 2013). The role of AI-2 to regulate biofilm formation has been recognized in various species (Yang, 2011), but its function in *Z. mobilis* has not been investigated.

**FIGURE 1:**
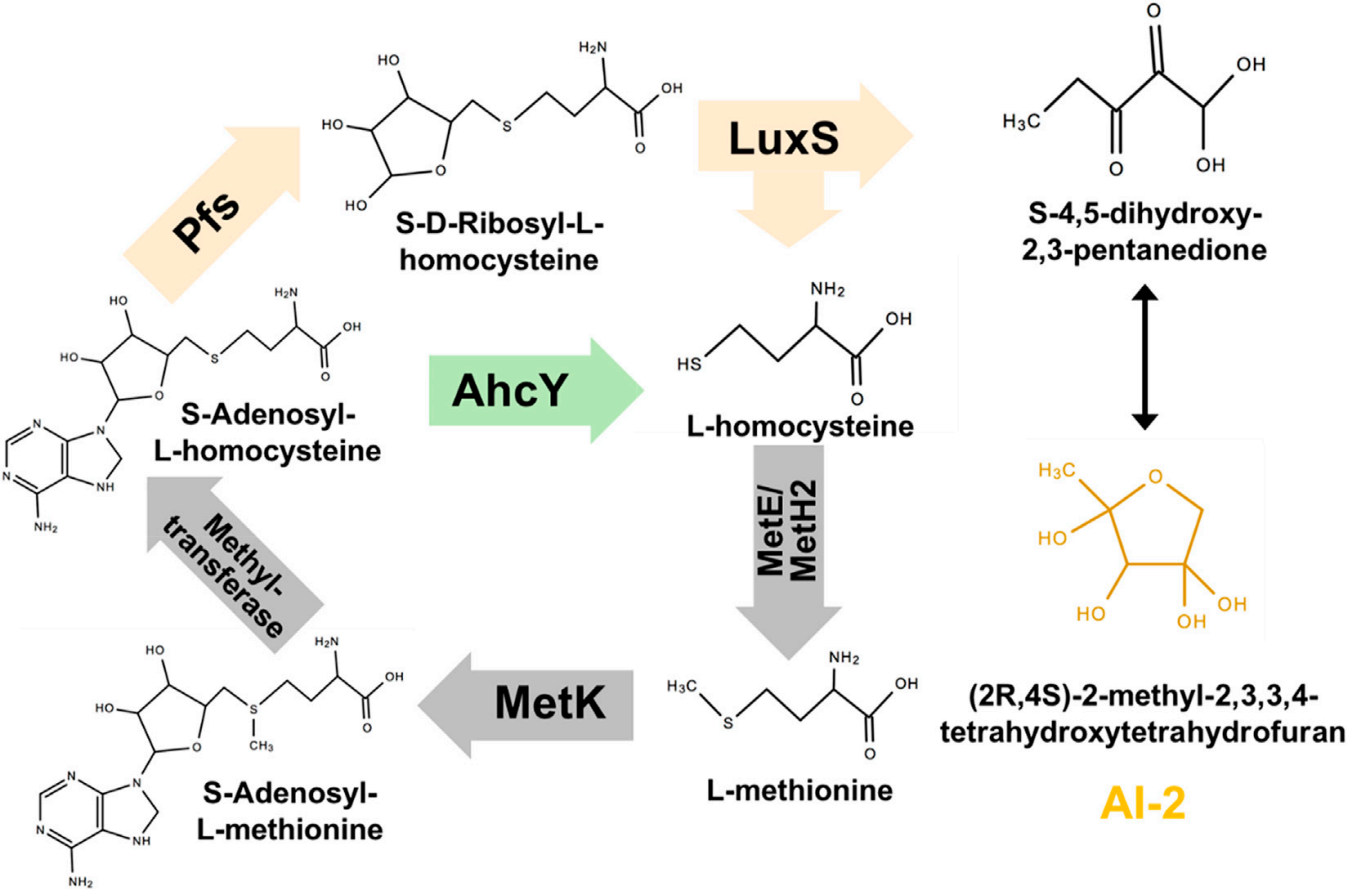
Constructing exogenous pathway for AI-2 synthesis in *Z. mobilis* ZM4. The orange genes were heterologously expressed in ZM4. The green gene should be deleted.

The synthesis pathway of AI-2 is conserved among all microorganisms: as shown in Figure 1, AI-2 is generated by S-ribosylhomocysteinase (LuxS) from S-ribosylhomocysteine (SRH), which is produced by 5′-Methylthioadenosine/S-adenosylhomocysteine nucleosidase (Pfs) from S-Adenosyl-L-homocysteine (SAH) (Pereira et al., 2013). This two-step synthetic pathway of AI-2 is also a part of cysteine and methionine circulation, existing in *Gammaproteobacteria*, *Betaproteobacteria*, *Epsilonproteobacteria*, *Spirochaetes*, *Actinobacteria*, *Firmicutes*, and *Deinococcus*-*thermus* (Sun et al., 2004). Instead of the two-step way conducted by Pfs and LuxS, *Z. mobilis* uses one-step hydrolysis to break up S-Adenosyl-L-homocysteine (SAH) and to complete the methyl cycle, making the cell unable to generate AI-2 (Yang et al., 2009; Yang et al. 2018).

In this work, we constructed the AI-2 synthetic pathway in *Z. mobilis* by heterologous expressing *pfs* and *luxS* from *E. coli* K12 (MG1655). Although the reconstructed strain successfully synthesized AI-2, its function in promoting the biofilm formation was not observed. Surprisingly, expression of only *pfs* significantly enhanced biofilm formation, which enhanced stress tolerance of strain to acetic acid, a major inhibitor in the lignocellulosic hydrolysate.

## 2 Materials and methods

### 2.1 Strains, media, and cultivation

All strains used in this study are listed in Table 1. Wildtype ZM4 was purchased from American Type Culture Collection (ATCC) and cultivated in RMG (10 g/L yeast extract, 20 g/L glucose, and 2 g/L KH_2_PO_4_) medium. For seed culture preparation, one colony of *Z. mobilis* was picked up from a solid plate and inoculated into a 5 mL RMG. After 24 h cultivation, 1 mL OD_600_ = 1.5 cultures was transferred into 100 mL RMG and cultivated statically at 30°C. The medium for transformant strain was supplied with 20 mg/L tetracycline. *E. coli* DH5α and *E. coli* JM110 applied in this work were used for the construction and propagation of plasmids carrying genes with and without methylation, respectively, which were grown in 5 mL Luria-Bertani (5 g/L yeast extract, 10 g/L tryptone, 10 g/L sodium chloride) of 15 mL tubes at 37°C, 200 rpm. *V. fisheri* BB152 and *V. fisheri* BB170, donated by Prof. Xiangan Han (Shanghai Veterinary Research Institute, Chinese Academy of Agricultural Sciences) and Prof. Bonnie Bassler (Princeton University), were recovered in MB (Marine Broth 2216) and cultivated in AB (Autoinducer Bioassay) medium (2.0 g/L casamino acids, vitamin-free, 12.3 g/L magnesium sulfate heptahydrate, 17.5 g/L sodium chloride, 174 mg/L L-arginine, 1% (v/v) glycerol and 10 mM potassium phosphate) for AI-2 detection.

**TABLE 1:** Plasmids used in this work.

Strains/Plasmids	Description
*E. coli* DH5α	*lacZΔM15, recA1*
*E. coli* JM110	*rpsL, dam-, dcm-*
*Z. mobilis* ZM4	ATCC31821
ZM4/pHW20a	ZM4 engineered with the vector pHW20a
ZM4/20a::pfs	ZM4 engineered with the overexpression of pfs from MG1655
ZM4/20a::luxS	ZM4 engineered with the overexpression of luxS from MG1655
ZM4/20a::pfs-luxS	ZM4 engineered with the overexpression of pfs and luxS from MG1655
*E. coli* K12 MG1655	ATCC 47076
*Vibrio fisheri* BB152	ATCC BAA-1117
*Vibrio fisheri* BB170	ATCC BAA-1119
pHW20a::P*gap*	pHW20a containing P*gap* from ZM4
pHW20a::pfs	pHW20a containing *pfs*of MG1655 driven by P*gap* ^*Zm*^
pHW20a::luxS	pHW20a containing *luxS* of MG1655 driven by P*gap* ^*Zm*^
pHW20a::pfs-luxS	pHW20a containing pfs and luxS of MG1655 driven by PgapZm
pEX18Tc	gene replacement vector with multi clone sites from pUC18
pEX18Tc𝚫0182	pEX18Tc containing recombinant sequences flanking ZMO0182

### 2.2 Construction of AI-2 synthetic pathway

Pfs can catalyze SAH to SRH, and LuxS splits SRH to L-homocysteine and S-4,5-dihydroxy-2,3-pentanedione (auto-cyclizing into AI-2) (Figure 1). With the transformation of *pfs* and *luxS*, *Z. mobilis* can recover methionine and produce AI-2 simultaneously. SAH hydrolase AhcY (ZMO0182) in *Z. mobilis* hydrolyzes SAH to L-homocysteine directly without AI-2 production. Knock out of AhcY was conducted to redistribute more carbon flux into the constructed pfs-luxS pathway.

### 2.3 Construction and identification of transformants

Plasmids and primers used in this study are given in Tables 1, 2. For heterologous gene expression, the *gap* ^*Zm*^ promoter was amplified by PCR and ligated with shuttle vector pHW20a in advance. Target genes were then amplified by PCR and infused with linearized vectors with P*gap* ^*Zm*^ by seamless cloning (Seamless Cloning master mix, Sangon Biotech, Shanghai, China), followed by propagating into *E. coli* DH5α. Due to the Restriction-Modification systems in *Z. mobilis*, the confirmed plasmids by PCR and Sanger sequencing were further transformed into *E. coli* JM110 for demethylation. The demethylated plasmids were then extracted from *E. coli* JM110 and electro-transformed into ZM4 by the Gene Pulser (Gene Pulser Xcell™, Bio-Rad). Electro-transformation was done with 1 mm gap cuvettes operated at 1.8 kV. Colonies were selected by tetracycline and confirmed by PCR.

**TABLE 2:** Primers used in this work.

Primer	Sequence (5′-3′)	Description
pfs-F	aat​aag​tta​gga​gaa​taa​acA​TGA​AAA​TCG​GCA​TCA​TTG​GTG​CAA	Amplify *pfs* for construction of 20a::pfs
pfs-R	cta​gag​gat​ccc​cgg​gta​ccT​TAG​CCA​TGT​GCA​AGT​TTC​TGC​AC	
luxS-F	aat​aag​tta​gga​gaa​taa​acA​TGC​CGT​TGT​TAG​ATA​GCT​TCA​CAG	Amplify luxS for construction of 20a::luxS
luxS-R	cta​gag​gat​ccc​cgg​gta​ccC​TAG​ATG​TGC​AGT​TCC​TGC​AAC​TTC	
pfs-F	aat​aag​tta​gga​gaa​taa​acA​TGA​AAA​TCG​GCA​TCA​TTG​GTG​CAA	Amplify pfs and luxS for construction of 20a::pfs-luxS
pfs (-luxS)-R	TTA​GCC​ATG​TGC​AAG​TTT​CTG​CAC	
(pfs-)luxS-F	AGA​AAC​TTG​CAC​ATG​GCT​AAA​TGC​CGT​TGT​TAG​ATA​GCT​TCA​CAG	
luxS-R	cta​gag​gat​ccc​cgg​gta​ccC​TAG​ATG​TGC​AGT​TCC​TGC​AAC​TTC	
0182-HI-F	ttg​cat​gcc​tgc​agg​tcg​act​cta​gaC​GAA​GGC​AGG​CTG​CCC​CTG​C	Amplify homologous fragments flank ZMO0182 for construction of pEX18Tc𝚫ZMO0182
0182-H1-R	AGC​AAG​GCT​GAT​GTC​ACG​GA	
0182-H2-F	TCC​GTG​ACA​TCA​GCC​TTG​CTG​GAT​CAT​TAT​CGT​TAT​TGA​TT	
0182-H2-R	aat​tcg​agc​tcg​gta​ccc​ggg​gat​ccT​CGC​TAC​CCG​CGC​TTA​TGT​C	

Gene deletion was performed by homologous recombination with the suicide vector pEX18Tc bearing tetracycline selection marker and sucrose counter-selection marker. Briefly, 500–1,000 bp fragments flanking ZMO0182 were amplified, and fused by seamless cloning as described above, which was propagated in *E. coli* DH5α, and confirmed by PCR and sequencing. The correct plasmid was transformed into *E. coli* JM110 to demethylation. Demethylated plasmid was electro-transformed into ZM4 as described above. The positive selection was screened using RMG supplemented with tetracycline after the first crossover recombination, followed by the second crossover recombination. A mutant with the gene deleted should be selected through the counter-selection using the rich medium supplemented with sucrose (Xia et al., 2019).

### 2.4 AI-2 detection

The process to detect AI-2 was modified based on previous work (Taga and Xavier, 2011). Briefly, the seed culture of *Z. mobilis* was inoculated into RMG with starting OD_600_ at 0.015 and cultivated at 30°C, 150 rpm for 18 h to enter the post-exponential phase when the most abundant AI-2 could accumulate. Triplicate samples, each with 4 mL, were centrifuged by Xiangyi H1650-W at 5,000 rpm for 5 min to collect the supernatant and passed it through a 0.22 μm sterile syringe filter. *Vibrio harveyi* BB170 was inoculated in a 15 mL flask with 5 mL AB medium overnight at 28°C, 150 rpm, and then diluted with AB medium at 1:5,000 as the seed culture. 0.2 mL supernatant and 1.8 mL seed culture were mixed and cultivated at 28°C, 150 rpm for AI-2 detection. The mixture’s bioluminescence intensity was measured after 6 h by Multimode Plate Reader (PE & ENSPIRE 2300, Perkin-Elmer, United States) to indicate AI-2 concentration. Fresh AB medium was used to replace the sample as the negative control, and synthetic AI-2 (D060111, Omm Scientific) or supernatant of *V. Harveyi* BB152 were applied as the positive controls. Considering the inhibition of tetracycline on growth and bioluminescence of BB170 during AI-2 detection and the high stability of the pHW20a plasmid (Dong et al., 2011), all transformants were cultivated without antibiotic for supernatant collection.

### 2.5 Motility ration

Bacterial motility was displayed as the motility ratio, which reflected a height ratio of the turbid part in the whole medium in a tube. Briefly, the seed culture of *Z. mobilis* was inoculated into 5 mL RMG of 15 mL tubes and cultivated at 30°C statically. Cells with weak motility would settle down due to the gravity, while cells with high motility will swim through the entire tube to remain turbid. After 24 h, the height of the whole media and the transparent portion were measured. The motility ratio was calculated as (1- the height of the transparent part/the height of the entire medium).

### 2.6 Quantitative analysis of biofilm

For biofilm establishment, plastic (Polymethyl Methacrylate, PM) flake at 1 cm × 1 cm was chosen as the abiotic platform. 24-wells plate with PM flakes pre-loaded was inoculated with seed culture and incubated at 30°C statically for cell attachment. Biofilm development was visualized and quantified by crystal violet staining. After cultivation in wells for 3 days, plastic flakes with biofilm formed were picked out and soaked in 1% crystal violet for 20 min, followed by rinsing with deionized water twice. The remaining biofilm with crystal violet staining was dissolved in 1 mL 95% ethanol and measured at OD_595_ by spectrophotometer (Multiskan GO 1510, ThermoFisher, Finland). Biomass in 24-wells plate was collected by resuspension and then measured at OD_600_ by spectrophotometer.

### 2.7 Toxicity study

Acetic acid toxicity for biofilms was studied by LIVE/DEAD™ BacLight™ Bacterial Viability Kit (L7012, Invitrogen, Thermo Fisher Scientific, United States). Based on previous work (Li et al., 2006), cells grown on plastic flakes in wells for 3 days to form mature biofilm and then incubated at 21 g/L acetic acid for 0.5 h. Then flakes with biofilm were picked out and rinsed in sterile H_2_O twice for being stained with the LIVE/DEAD Bac Light™ Bacterial Viability and Counting Kit, followed by examination with Super-resolution Multiphoton Confocal Microscope (TCS SP8 STED 3X, Leica, Germany).

### 2.8 Test of stress tolerance

10 mL OD_600_ = 1.5 seed culture was inoculated into 90 mL RMG. The culture was cultivated at 30°C, 150 rpm for 12 h to measure OD_600_. The strains’ tolerance to 7.5% ethanol, 2.1 g/L acetic acid (density: 1.05 g/mL), or 1 g/L vanillin were evaluated by comparing with OD_600_ of cells cultivated without inhibitor. Transformants were supplemented with 20 mg/L tetracycline.

## 3 Results and discussion

### 3.1 Growth of strains with heterologous gene expression

**FIGURE 2:**
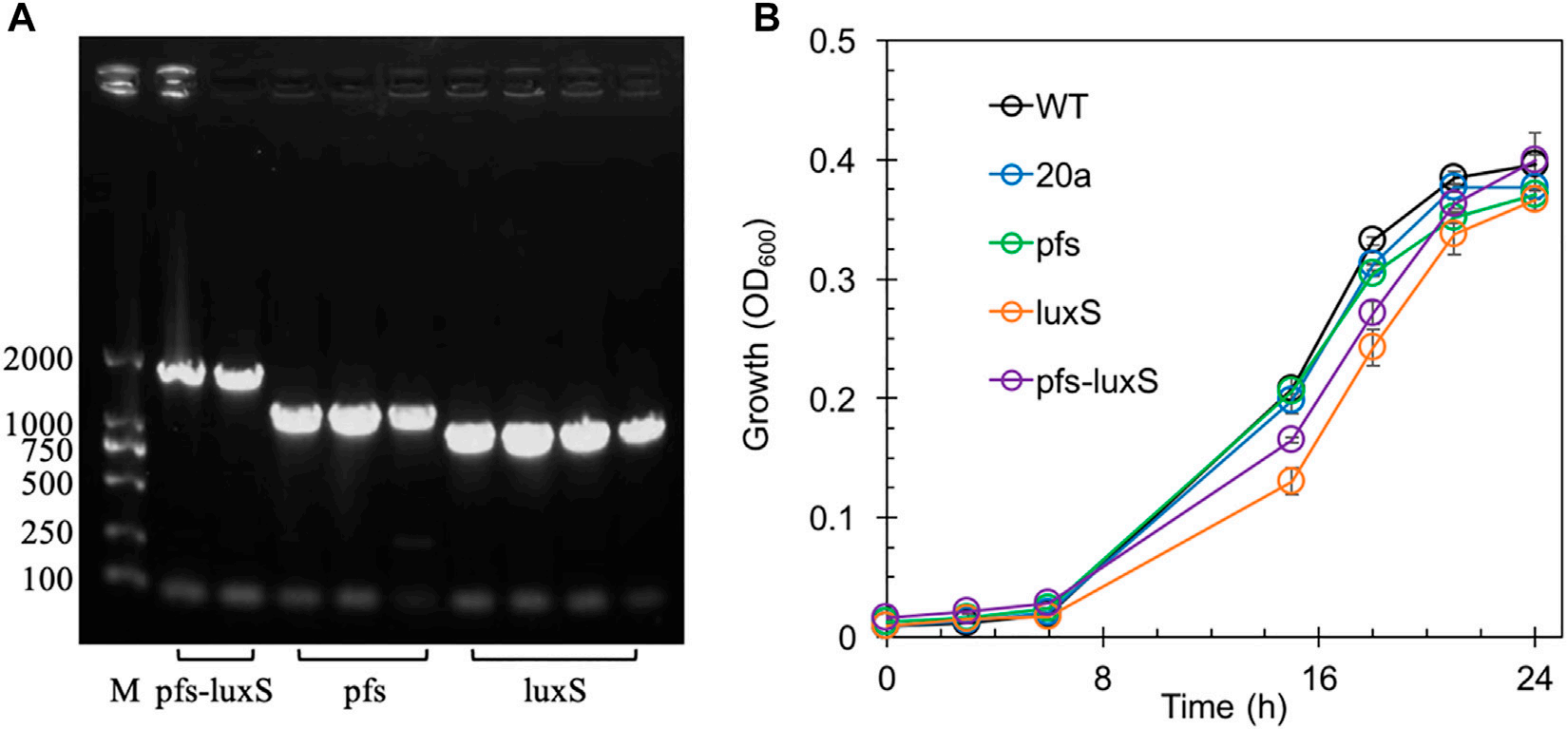
The confirmation of genes manipulation **(A)** and growth profiles of transformants and wildtype *Z. mobilis* **(B)**. M stands for DNA marker DL 2000.

Transformants with heterologous expression of *pfs* and *luxS* were selected by tetracycline screen and PCR confirmation. Figure 2A showed the correct length of bands obtained by colony PCR of transformants, hinting that *pfs* or/and *luxS* were successfully transformed into ZM4. Knock out of AhcY was also conducted to enhance the Pfs-LuxS pathway. However, no transformant with AhcY deletion was obtained, which might because S-adenosyl-L-homocysteine hydrolase AhcY is essential for *Z. mobilis* to complete the cysteine and methionine circulation and prevent the accumulation of toxicant SAH in wildtype. To elucidate the effects of Pfs and LuxS on growth, the biomass of strains was evaluated before the investigation of other physiological performances. As visualized in Figure 2B, ZM4:luxS and ZM4:pfs-luxS had a slower growth rate in the log phase (6–20 h) when compared with WT and ZM4:20a, which suggested that the expression of *luxS* might cause a metabolic burden on bacteria by plasmid maintenance and heterologous gene expression (Pasini et al., 2016). However, heterologous expression of *pfs* leads to almost no metabolic burden in terms of growth rate, which may occur due to the clearance of toxic SAH by Pfs (Parveen and Cornell, 2011). All strains approached similar OD_600_ at stationary phase 24 h, indicating that the side effect of heterologous expression of *pfs* and/or *luxS* was negligible at the end of growth (less than 5%), eliminates the requirement for considering the metabolic burden in the following experiments.

### 3.2 AI-2 produced by expression of *pfs* and *luxS*

**FIGURE 3:**
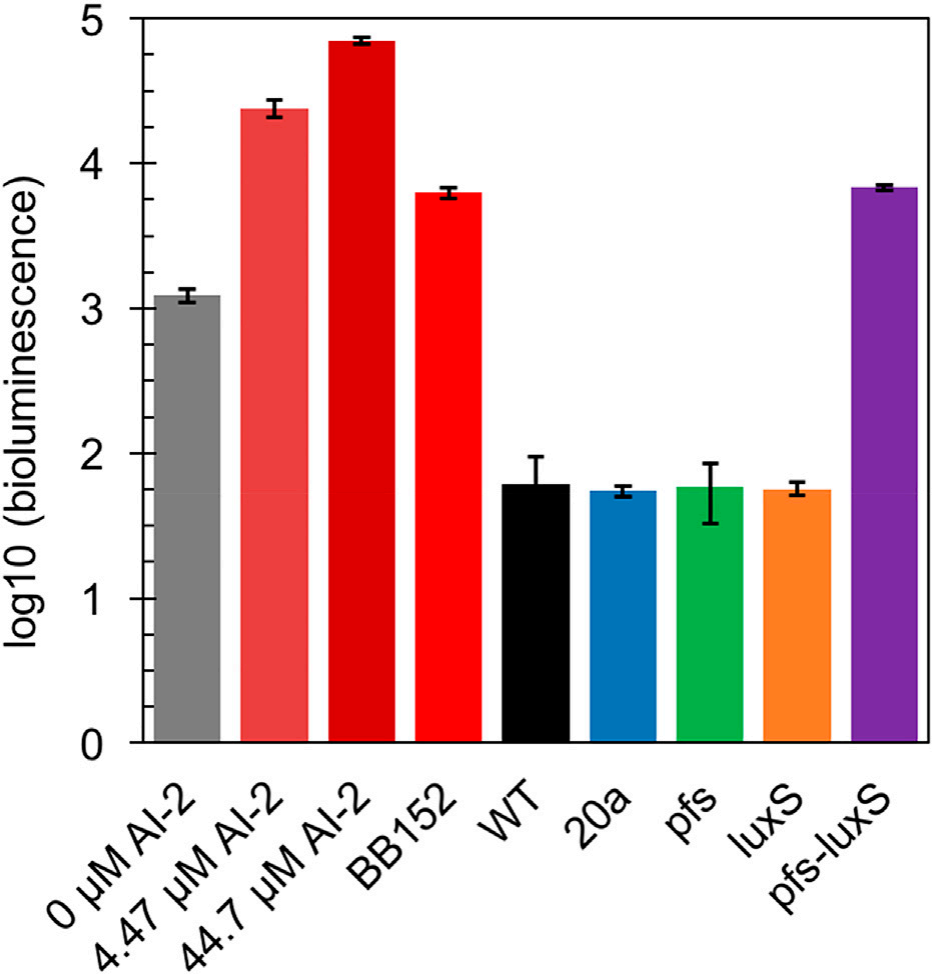
Bioluminescence assay by *V. fisheri* BB170 for AI-2 semi-quantification in the supernatant of cultures.

In principle, ZM4 with *pfs* and *luxS* heterologous expression could produce AI-2 even though with the failure deletion of ZMO0182. The bioluminescent density of *V. fisheri* BB170 was used to semi-quantify AI-2 when taking AI-2 and *V. fisheri* BB152 supernatant as positive controls and AB medium as the negative control. As shown in Figure 3, the AB medium without AI-2 allowed BB170 to present the basal bioluminescence. The density of bioluminescence positively correlated with the concentration of AI-2. Based on the relation, the AI-2 in BB152 supernatant should go below 4.47 μM. Interestingly, the supernatant of *Z. mobilis* strains except for ZM4:pfs-luxS pulled down the bioluminescence of BB170, making it lower than the negative control, which was reasonable as the metabolites of *Z. mobilis*, such as major product ethanol, possibly played an inhibitory role on bioluminescent. Even though, the supernatant of ZM4:pfs-luxS lead to a higher bioluminescence intensity than the negative control and other *Z. mobilis* strains, which demonstrated that AI-2 was successfully produced and the functions of Pfs and LuxS can be confirmed in *Z. mobilis*.

### 3.3 Motility strengthened by *pfs* in an AI-2 independent way

**FIGURE 4:**
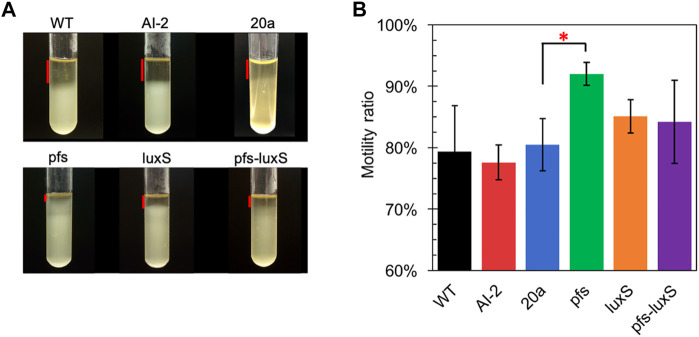
Motility test of *Z. mobilis* strains by settlement method **(A)** The cell sedimentation in the tubes **(B)** Motility ratio = 1-the height of the transparent part/the height of the entire medium. WT: wildtype. All transformants are constructed from ZM4. Three replicates. * represents *p*-value < 0.1.

Many studies regarding AI-2-mediated quorum sensing have confirmed its role in motility, which includes swimming motility and swarming motility (Teren et al., 2018; Wang et al., 2018). The test of cell motility using static cultivation in tubes reveals the relationship between quorum sensing and the motility of ZM4. The sedimentation of cells during static cultivation can be interfered with by the vigorous motility of *Z. mobilis*, resulting in an unevenly distributed transparent medium in the upper part and turbid culture in the lower part (Figure 4).

Figure 4 showed that wildtype owned a low motility ratio, which was not changed, even adding 4.47 μM AI-2, which was straight evidence to confirm the invalidation of AI-2 on the mobility of *Z. mobilis*. Comparing with wildtype, ZM4:20a showed no statistically different motility, but the heterologous expression of *pfs* strengthened cell motility. Interestingly, neither ZM4:luxS nor ZM4:pfs-luxS performed significantly enhanced mobility, even though the AI-2 produced by ZM4: pfs-luxS, reflected that the mobility of *Z. mobilis* depends on other specific reactions catalyzed by Pfs, instead of QS signal molecules AI-2. Pfs promotes SAM-dependent transmethylation by reducing SAH, simultaneously yield methylated DNA, RNA, and protein (Parveen and Cornell, 2011). Considering DNA methylation could regulate expression of motility-related genes (Vandenbussche et al., 2020), it is reasonable that strain with heterologous expression of *pfs* exhibited the strongest motility. ZM4:pfs-luxS also expressed Pfs and showed slightly enhanced motility but with insignificance, which might arise due to the relatively low expression of *pfs* as it was co-expressed with *luxS*.

### 3.4 Biofilm enhanced by *pfs* in an AI-2 independent way

**FIGURE 5:**
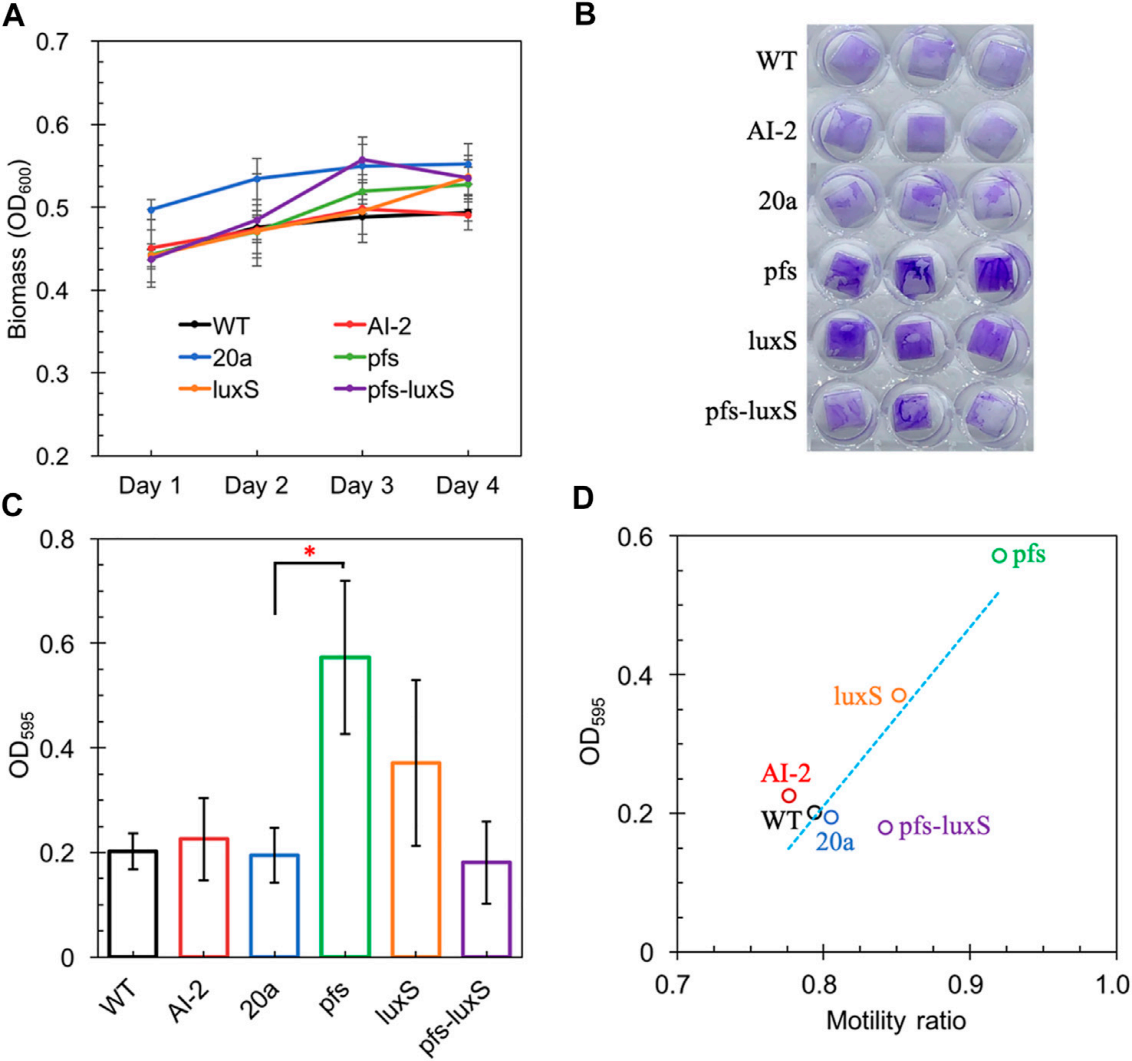
The Growth of strains on a 24-wells plate **(A)**; Crystal violet staining of WT, WT (with 4.47 μM AI-2 addition), ZM4:20a, ZM4:pfs, ZM4:luxS, and ZM4:pfs-luxS **(B)**; Quantification of biofilm **(C)**; Correlations between biofilm and bacterial motility **(D)**. Three replicates. * represents *p*-value < 0.1.

Altered motility usually affects biofilm formation (Tolker-Nielsen, 2015; Jani et al., 2017). AI-2 has been verified to promote cell aggregation and biofilm formation in various bacteria (Wang et al., 2014; Laganenka et al., 2016; Mizan et al., 2016), but its role in *Z. mobilis* remains elusive. *Z. mobilis* exhibited greater attachment and improved biofilm formation on the hydrophobic surface than on hydrophilic glass in this study. The biofilm of wildtype and AI-2 producing strain ZM4:pfs-luxS were quantified to investigate the role of AI-2 on biofilm formation. As shown in Figure 5A, bacteria kept growing in the first 3 days and then entered the stationary phase. Thus, the third day with the highest OD_600_ was chosen for biofilm quantification.

Figure 5 shows a comparable quantity of biofilm formed by strains with or without AI-2, which reflects that the exogenous addition of AI-2 was incapable of contributing to biofilm formation in *Z. mobilis*. Moreover, the biofilm formation of ZM4:pfs-luxS and ZM4:20a were at the same level, presenting that the endogenous production of AI-2 did not correlate with the biofilm formation of *Z. mobilis*. ZM4:pfs produced the most abundant biofilm, which suggested that the product of Pfs might play a critical role in biofilm formation in *Z. mobilis*. The large error bar in Figure 5C indicates that the biofilm of *Z. mobilis* was not as tight as other bacteria biofilms such as *Pseudomonas aeruginosa* or *Bacillus subtilis*, which could be easily removed through rinsing (Figure 5B) (Chua et al., 2014; Duanisassaf et al., 2016). Researches on *E. coli* revealed that the cell’s high motility contributes to forming surface-adherent structures (Jani et al., 2017). Combining this issue with the previous motility evaluation, since adherence of cells to the plastic surface is induced by physical contact, ZM4:pfs with higher motility hold a greater probability of contact to the plastic surface in more biofilm formation. Plus, methylated DNA has been reported to play an essential role in efficient biofilm formation (Aya Castañeda et al., 2015) which could explain the increased volume of biofilm formed by ZM4:pfs.

Typically, biofilm formation consists of five steps: attachment, aggregation, maturation, full development, and dispersion (Verderosa et al., 2019). The first step when cells migrate and adhere to an abiotic surface is severely affected by motility, which is in line with the strong positive correlation between biofilm formation and motility observed in our work (Figure 5D), hinted this step might be the most influential one during biofilm formation by *Z. mobilis*.

### 3.5 Stress tolerance of *Zymomonas mobilis* strengthened by biofilm

**FIGURE 6:**
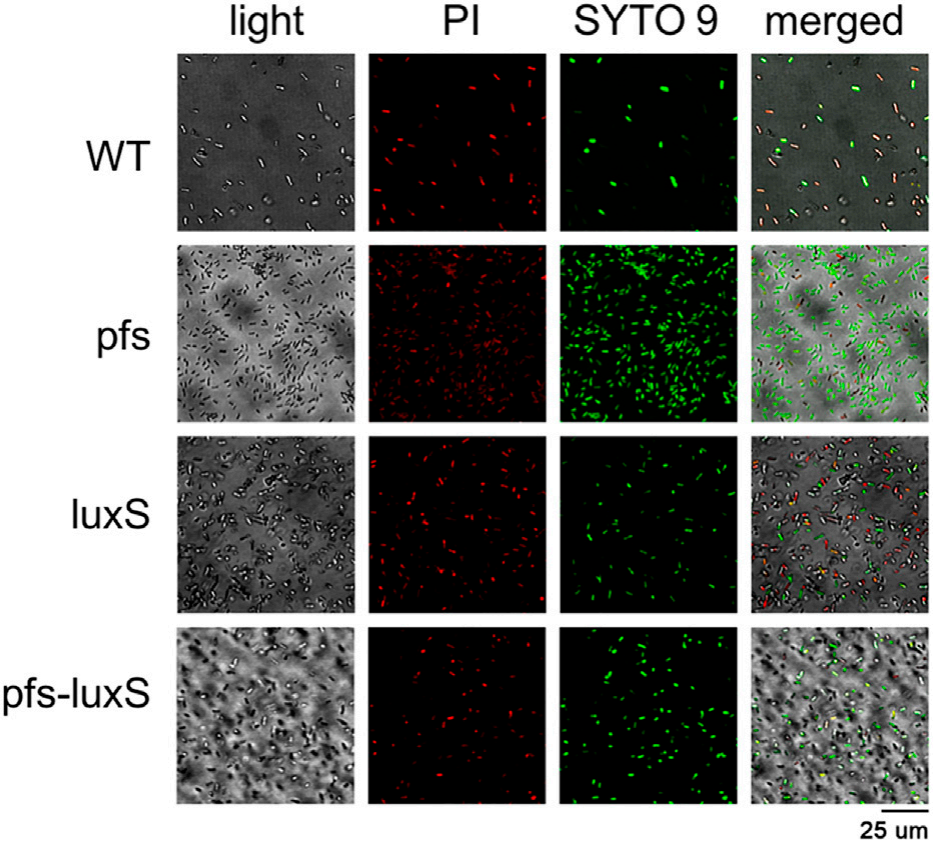
Images of WT, ZM4:pfs, ZM4:luxS, ZM4:pfs-luxS after 2.1 g/L acetic acid shock. SYTO 9 stain and propidium iodide were used to stain cells. Live cells fluoresced green and dead cells fluoresced red.

Biofilm formation has been proven to enhance stress tolerance in several *Z. mobilis* strains (Li et al., 2006; Todhanakasem et al., 2018). Studies have confirmed that biofilm can help *Z. mobilis* be more tolerant to various inhibitors in lignocellulosic hydrolysate (Li et al., 2006; Todhanakasem et al., 2014). We measured the biofilm strains’ viability to test their stress tolerance to acetic acid, one of the significant lignocellulosic hydrolysate inhibitors. Figure 6 showed that the viability of ZM4:pfs was much higher than WT, which indicated increased biofilm formed by ZM4:pfs was helpful to resist the stress from inhibitor outside. The results verified the role of accumulated biofilm in improving tolerance of bacteria to inhibitors.

**FIGURE 7:**
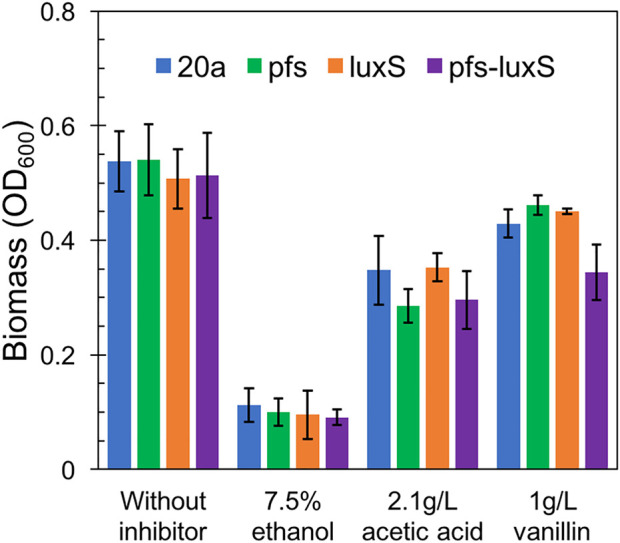
Growth of planktonic cells in flasks under ethanol, acetic acid, and vanillin addition, OD_600_ was measured at 12 h. The statistic *p*-value >0.05 among each group.

As ZM4:pfs had greater tolerance to acetic acid, but it remains uncertain whether this strengthens derived from the enzyme Pfs or the protection by biofilm. Fermentation with inhibitors was conducted to address this question. As Figure 7 showed, planktonic cells reached similar OD at 12 h without inhibitor in the rotated flask, whose hydrophobic glass surface would stop the biofilm formation. ZM4:pfs performed a similar growth with ZM4:pHW20a under the stressful conditions with ethanol, acetic acid, or vanillin supplementation. These results confirmed the improvement of stress tolerance in ZM4:pfs was due to an increase in the quantity of biofilm induced by Pfs instead of the product of protein Pfs.

### 3.6 The mechanism of pfs and LuxS on biofilm formation and stress tolerance

**FIGURE 8:**
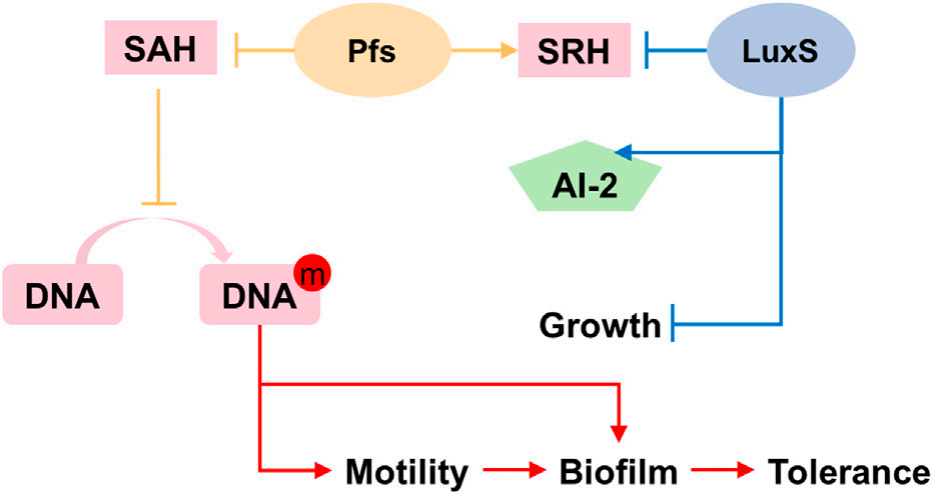
Proposed mechanism of Pfs and LuxS on AI-2 production, motility, biofilm formation, and stress tolerance.

Based on the results illustrated above, the mechanism underlying the heterologous expression of *pfs* and *luxS* is proposed. As Figure 8 shows, the co-expression of *pfs* and *luxS* enables ZM4 to produce AI-2. However, AI-2 does not affect the growth, motility, biofilm formation, or stress tolerance of ZM4. Unexpectedly, expression of *pfs* alone enhances cell motility and biofilm formation, thus strengthens strains’ stress tolerance, which might due to the accumulated methylated DNA because of the inhibition by low concentration of SAH that was utilized by Pfs (Aya Castañeda et al., 2015). Whereas, co-expression of *pfs* and *luxS* do not show similar results as the cysteine-methionine circulation is finished so that SAH cannot be maintained at low concentration and no extra methylated DNA could accumulate. Besides, Pfs can hydrolyze SAH into SRH, which might contribute to biofilm formation (Challan Belval et al., 2006). Finally, increased biofilm formation protects cells from inhibitors added exogenously, thus strengthened bacterial stress tolerance.

## 4 Conclusion

We constructed an AI-2 producing strain by heterologous expression of *pfs* and *luxS* with a negligible metabolic burden. However, neither cell motility nor biofilm formation was observed to be improved by either endogenously generated or exogenously added AI-2. Surprisingly, expressing *pfs* alone enhanced cell motility and biofilm formation, contributing to stress tolerance strengthen. This work verifies the importance of biofilm on environmental stresses and provides a new method to improve biofilm formation in *Z. mobilis*.

## Data Availability

The raw data supporting the conclusions of this article will be made available by the authors, without undue reservation.
